# Wave Electromechanical Coupling Factor for the Guided Waves in Piezoelectric Composites

**DOI:** 10.3390/ma11081406

**Published:** 2018-08-11

**Authors:** Yu Fan, Manuel Collet, Mohamed Ichchou, Olivier Bareille, Lin Li

**Affiliations:** 1 School of Energy and Power Engineering, Beihang University, Beijing 100191, China; fanyu04@buaa.edu.cn; 2 Beijing Key Laboratory of Aero-Engine Structure and Strength, Beijing 100191, China; 3 Laboratoire de Tribologie et Dynamique des Systemes, Ecole Centrale de Lyon, 69130 Ecully, France; manuel.collet@ec-lyon.fr (M.C.); mohamed.ichchou@ec-lyon.fr (M.I.); olivier.bareille@ec-lyon.fr (O.B.)

**Keywords:** guided waves, wave electromechanical coupling factor (WEMCF), piezoelectricity composites, wave and finite element method, reduced model

## Abstract

A novel metrics termed the ‘wave electromechanical coupling factor’ (WEMCF) is proposed in this paper, to quantify the coupling strength between the mechanical and electric fields during the passage of a wave in piezoelectric composites. Two definitions of WEMCF are proposed, leading to a frequency formula and two energy formulas for the calculation of such a factor. The frequency formula is naturally consistent with the conventional modal electromechanical coupling factor (MEMCF) but the implementation is difficult. The energy formulas do not need the complicated wave matching required in the frequency formula, therefore are suitable for computing. We demonstrated that the WEMCF based on the energy formula is consistent with the MEMCF, provided that an appropriate indicator is chosen for the electric energy. In this way, both the theoretical closure and the computational feasibility are achieved. A numerical tool based on the wave and finite element method (WFEM) is developed to implement the energy formulas, and it allows the calculation of WEMCF for complex one-dimensional piezoelectric composites. A reduced model is proposed to accelerate the computing of the wave modes and the energies. The analytical findings and the reduced model are numerically validated against two piezoelectric composites with different complexity. Eventually an application is given, concerning the use of the shunted piezoelectric composite for vibration isolation. A strong correlation among the WEMCF, the geometric parameters and the energy transmission loss are observed. These results confirm that the proposed WEMCF captures the physics of the electromechanical coupling phenomenon associated with the guided waves, and can be used to understand, evaluate and design the piezoelectric composites for a variety of applications.

## 1. Introduction

The concept of smart structures provides another promising possibility of solving a variety of engineering issues, such as vibration control, noise reduction, fault diagnose, wireless sensing, self powering and so on. Smart structures could be defined as ‘the structures that can sense external disturbance and respond to that in a desired fashion’ [1]. Generally a smart structure can be implemented by integrating some ‘smart materials’ that couple at least another field to the mechanical field. This introduces new design variables to modify or measure the mechanical characteristics. An additional subsystem can then be designed regarding the new variables so as to have the coupled structural system work in a desired manner. These smart materials involve electrorheological (ER) materials, magnetorheological (MR) materials, magnetostrictive materials, electrostrictive materials, shape memory alloys (SMA), piezoelectric materials and so on. Among them all, the piezoelectric materials have the advantages of light-weight, large working frequency range and high power density, therefore they are undoubtedly the most mature and those with the most applications [2].

Piezoelectric effect that couples the electric and mechanical fields is the corner stone of all the piezoelectric-based smart structures. It naturally comes into a question that how to quantitatively describe the ’strength’ of the electromechanical coupling induced by the piezoelectric materials. The criteria that can measure the converting capability or the coupling strength are termed the ‘electromechanical coupling factors’ (EMCF). In general, there are two scales regarding EMCF. The first one is the material scale [3]. EMCF in this scale is used to describe the coupling strength or converting capability of a piezoelectric material under certain uniaxial stress situations. EMCF in this scale is therefore directly related to the material parameters. For instance for the thickness stretch deformation (referred to as the ‘13’ mode), the coupling factor $k_{31}^{2}=d_{13}^{2}/\left(\epsilon_{33}^{T}c_{11}^{D}\right)$, where $d_{13}$, $\epsilon_{33}^{T}$ and $c_{11}^{D}$ are all material parameters [4]. Likewise, terms $k_{33}^{2}$, $k_{51}^{2}$ can also be obtained for the longitudinal (the ‘33’ mode) and torsional (the ‘51’ mode) deformation respectively. These terms are also called static coupling factors. They allow one to choose an appropriate piezoelectric material before it is manufactured into certain components. They are especially useful in actuating and sensing [5,6] where a uniaxial tress field is assumed.

If we consider the host structure and the piezoelectric materials/transducers as a whole, and concern the dynamic behaviour, the EMCF should be defined in the structural scale. A widely recognized approach is to define the EMCF $k_{d}^{2}$ based on natural modes [4,7], by

(1) $$k_{d}^{2}=\frac{\omega_{r}^{2}-\omega_{a}^{2}}{\omega_{r}^{2}}$$

where $\omega_{r}$ and $\omega_{a}$ are the resonance and anti-resonance frequencies in the frequency response fuction (FRF) of V/Q respectively; *V* and *Q* are the voltage and charge of the piezoelectric materials respectively. Moreover, $\omega_{r}$ is also the open-circuit frequency of a mode and $\omega_{r}$ is also the short-circuit modal frequency [8]. Term $k_{d}^{2}$ is therefore called modal electromechanical coupling factor (MEMCF) [9] as well. Theoretically speaking, arbitrary deformation of a finite structure can be expressed as the superposition of the modal deformations, and the modes whose natural frequencies are close to the considered excitation frequency range will have dominant contribution. In this regard, it is reasonable to define the EMCF in terms of modes. Please obtaining the MEMCF is very convenient both experimentally and numerically because only two set of modal analysis results are required. MEMCF as a metrics in the structural scale has many important applications. A direct application is the design of modal transducers [8,10], where the MEMCF is used to examine whether the piezoelectric subsystem is coupled only with the target mode (modal sensor) or it is uncoupled with the target mode(modal filter). Another application is the passive vibration control. Thomas et al. [9] found, via closed-form expressions, that the best modal damping induced by the resistor and resistor-inductor shunts depend only on the MEMCF once the structural parameters remain constant. This indicates that MEMCF can be used as a criterion to optimize the geometrics of the piezoelectric materials [11,12].

Alternatively, the structural deformation can also be described by the superposition of wave motions, while natural modes are understood as standing waves induced by the reflection of waves on the boundaries [13,14,15]. The wave perception is especially suitable in dealing with high-frequency or transient vibration problems, where many modes are involved and the resonance behaviour is less apparent. Many piezoelectric strategies have been proposed based on the wave perception. For example, the periodically distributed piezoelectric materials with identical shunting circuits are used to modify the wave properties in the interested frequency band so as to dissipate or localize the injected energy [16,17,18,19]. Moreover, piezoelectric materials are designed as actuators and sensors for the waves in cylinders [20,21] and plates [22,23,24] for structural health monitoring (SHM) purpose.

In the wave perception, the EMCF should quantify the coupling strength between the mechanical and electric fields during the passage of a wave in piezoelectric composites, and in this paper it is termed the ‘wave electromechanical coupling factor’ (WEMCF) to distinguish itself from the aforementioned coupling factors. Only a few works have been devoted to this subject, and WEMCF is defined by different means. Chen et al. [25] calculated WEMCF by $k=\left(V_{\mathrm{oc}}-V_{\mathrm{sc}}\right)/V_{\mathrm{sc}}$ for Rayleigh-type surface acoustic waves in a semi-infinite with alternating piezoelectric and non-piezoelectric super-lattices, where $V_{\mathrm{oc}}$ and $V_{\mathrm{sc}}$ are the group velocity in open-circuit and short-circuit situation respectively. Fan et al. [26] used the Green’s function method to calculate the WEMCF for a Lamb wave in a multi-layered plate. The authors [27] defined the WEMCF as the proportion of the electric energy associated with the block capacitance in elastic energy. We showed that the WEMCF can be used to decouple the geometric design from the electric design for the one-dimensional piezoelectric waveguide, and it allows to achieve the desired band gaps by using a minimum amount of piezoelectric materials.

The existing studies of WEMCF raise two questions: (1) Is the proposed WEMCF consistent with the MEMCF? and (2) How to build a general numerical tool for the calculation of WEMCF for complex piezoelectric composites? Since a structural dynamics problem is always possible, at least in principle, to arrive at the same conclusions by either mode or wave approach (often referred to as ‘wave-mode duality’ [14,15]), a positive answer should be given of the first question to achieve theoretical closure. The second question should be addressed so that WEMCF can be used in real engineering applications.

In this paper, we further study the WEMCF for one-dimensional piezoelectric waveguide and address both the aforementioned questions. In Section 2 we propose a frequency formula to define the WEMCF. It is difficult to implement but it is naturally consistent with the MEMCF. Then we propose two energy formulas to approximate the frequency formula. Since the energy formulas are easier to implement, both the theoretical closure and the computational feasibility are achieved. To do that, we first outline the wave and finite element method (WFEM) in Section 3, including the basic procedures and a reduced model applicable for piezoelectric composites. In Section 4 the energy formulas are discussed in detail, including the definition, demonstration and implementations. In Section 5 the analytical findings and implementations are validated. It is also shown that the use of the reduced unit cell model can rapidly and accurately capture the WEMCF. Eventually, an application concerning the control of energy flow using shunted piezoelectric composite is presented in Section 6, in order to illustrate the usage of WEMCF.

## 2. Definitions of WEMCF

**Figure 1:**
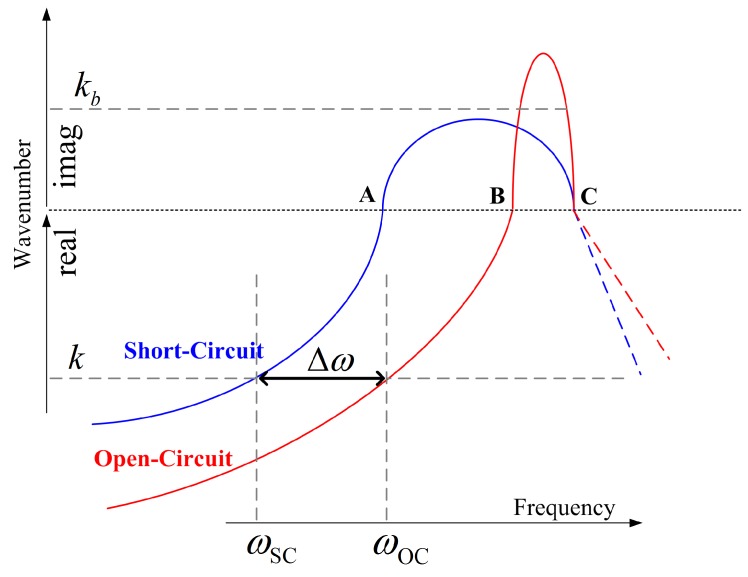
Illustration of dispersion curves for OC and SC status of a piezoelectric waveguide.

It is widely known that the equivalent stiffness of the piezoelectric materials will be changed when the electrodes are set to the short circuit (SC) or open circuit (OC) statuses. Consequently, a piezoelectric composite will exhibit different dispersion curves in SC and OC statuses [18], as illustrated in Figure 1. This inspires us to use the relative difference of the propagating frequencies of OC and SC statues to define the WEMCF $K_{W}^{2}$, as:

(2) $$K_{W}^{2}=\frac{\omega_{\mathrm{OC}}^{2}-\omega_{\mathrm{SC}}^{2}}{\omega_{\mathrm{SC}}^{2}}$$

where $\omega_{\mathrm{OC}}$ and $\omega_{\mathrm{SC}}$ are the propagating frequencies of the wave in the OC and SC statuses respectively. This equation is similar to Equation (1) which defines the MEMCF [4,8,28], but the frequencies terms have different physical means.

Please note that the wave shapes at the border frequencies of a band gap, namely A, B and C in Figure 1, are also the modal shapes of the piezoelectric composite under certain boundaries conditions [29]. This fact indicates that the coupling strength of these waves (A, B and C) can be given either by WEMCF or MEMCF. The WEMCF and MEMCF should give the same value at these frequencies, because a same deformation should be given a same value of its electromechanical feature, no matter the deformation is understood as a wave or a mode. In this case the travelling frequencies of the waves ($\omega_{\mathrm{OC}}$ and $\omega_{\mathrm{SC}}$) are exactly the natural frequencies ($\omega_{r}$ and $\omega_{a}$) of the modes. Hence Equations (1) and (2) yield the same value. This means that WEMCF defined by Equation (2) is consistence with MEMCF.

Definition of WEMCF by Equation (2) may be reasonable and intuitive. However, there are two main challenges. The first one is that Equation (2) works only for propagating waves, because band gaps may have different depth in OC and SC statuses, shown in Figure 1. Consequently it is difficult to link the evanescent waves between OC and SC, for example in Figure 1 the wave with wavenumber $k_{b}$ does not have its corresponding wave in the SC case. The second challenge is related to computational issues. For complex waveguide whose dispersion relations has veering and intersection (examples can be found in reference [30,31]), it will be difficult to correlate same type of propagating waves from the dispersion relations at OC and SC statues.

Alternatively, WEMCF can be defined by the fraction of energies at one electrode status:

(3) $$K_{1}^{2}=\frac{W_{e}}{V}$$

where $W_{e}$ is the electric energy and *V* is the mechanical energy. This definition is applicable for both evanescent and propagating waves. It only requires the dispersion curves at one situation so no efforts are needed to correlate the waves between OC and SC. Wave shapes have to be known to calculate the energies $W_{e}$ and *V*. In the following sections we will present different ways for the calculation of the energy terms in Equation (3) based on wave and finite element method (WFEM). The consistency with MEMCF will be demonstrated and validated.

## 3. Wave and Finite Element Method (WFEM)

Wave and finite element method (WFEM) [30,32,33,34] is a general numerical tool widely used to analyse the free and forced wave propagation in uniform and periodic structures. It requires only the modelling of the smallest repetitive substructure (termed the unit cell) of the whole structure, therefore is much faster than the conventional finite element method. WFEM yields the dispersion curves of the waves by solving the eigenvalue problem which can be formulated in many different ways [35]. In this paper, WFEM is developed to compute the required energy terms, and also as a general representation of the dynamics of the piezoelectric composite. The properties of the wave shapes derived from WFEM will be used later to demonstrate the consistency between the WEMCF and MEMCF. For these reasons we briefly outline the basic procedure of WFEM. A reduce model for the piezoelectric composite proposed earlier by the authors [31,36] is also outlined, and it will be used to accelerate the computing the energy terms.

### 3.1. Basic Procedure

**Figure 2:**
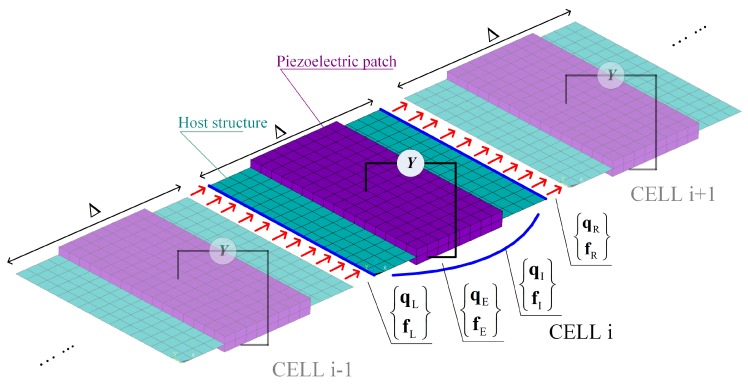
Illustration of the unit cells in a piezoelectric waveguide.

The first step of WFEM is to model one unit cell by finite element method. Any existing FEM package can be used, and in this paper ANSYS 17 is applied to generate the mesh and the required matrices of the unit cell modelling. All the following-up procedures of WFEM, including the solving of the eigenvalue problem, the reduced model, the post-processing are implemented by an in-house Python code developed by the authors. The Python code is validated against the analytical and full finite element method in many applications [30,31,36]. In absence of any external loads, the dynamic equations of a unit cell in the periodic piezoelectric structure write

(4) $$M\left(\begin{array}{c} {\ddot{q}}_{L}\\ {\ddot{q}}_{R}\\ {\ddot{q}}_{I}\\ {\ddot{q}}_{E} \end{array}\right)+K\left(\begin{array}{c} q_{L}\\ q_{R}\\ q_{I}\\ q_{E} \end{array}\right)=\left(\begin{array}{c} f_{L}\\ f_{R}\\ 0\\ 0 \end{array}\right)$$

where q is the displacement vector and f the force vector; K and M denote the generalized stiffness and mass matrices respectively; Subscripts L, R, I and E respectively refer to the left-side, right-side and internal mechanical Degree-of-freedoms (DOFs) and the electric voltage DOFs; A dot means derivation with respect to time *t*. The terms are also illustrated in Figure 2.

Specifically, the generalized mass matrix has the form of

(5) $$M=\left[\begin{array}{cccc} M_{\mathrm{LL}} & M_{\mathrm{LR}} & M_{\mathrm{LI}} & 0\\ M_{\mathrm{LR}}^{T} & M_{\mathrm{RR}} & M_{\mathrm{RI}} & 0\\ M_{\mathrm{LI}}^{T} & M_{\mathrm{RI}}^{T} & M_{\mathrm{II}} & 0\\ 0 & 0 & 0 & 0 \end{array}\right]$$

and the generalized stiffness matrix K writes

(6) $$K=\left[\begin{array}{cc} G & P\\ P^{T} & -C_{p}-Y \end{array}\right]=\left[\begin{array}{cccc} G_{\mathrm{LL}} & G_{\mathrm{LR}} & G_{\mathrm{LI}} & P_{\mathrm{LE}}\\ G_{\mathrm{LR}}^{T} & G_{\mathrm{RR}} & G_{\mathrm{RI}} & P_{\mathrm{RE}}\\ G_{\mathrm{LI}}^{T} & G_{\mathrm{RI}}^{T} & G_{\mathrm{II}} & P_{\mathrm{IE}}\\ P_{\mathrm{LE}}^{T} & P_{\mathrm{RE}}^{T} & P_{\mathrm{IE}}^{T} & -C_{p}-Y \end{array}\right]$$

where G and P are the mechanical stiffness and piezoelectric matrices respectively, $C_{p}$ the intrinsic capacitance matrix and Y external electric impedance. More details about the finite element model of a piezoelectric structure can be found in Benjeddou’s review [37].

The second step of WFEM is taking into account the periodicity. According to the Bloch theory, the wave of the form $e^{j\omega t-kx}$ that travels in the periodic structure should satisfy the condition

(7) $$q_{R}=\lambda q_{L}$$

where $\lambda =e^{-jk\Delta }$ describes the amplitude and phase change when the wave propagates from the left side to the right side of a unit cell. *k* is the wavenumber and Δ is the length of the unit cell along the propagation (periodicity) direction. Additionally, the equilibrium between the adjacent cells implies

(8) $$f_{R}=-\lambda f_{L}$$

Then the aim of the wave modal analysis is to find $q={\left(\begin{array}{cccc} q_{L}^{T} & q_{R}^{T} & q_{I}^{T} & q_{E}^{T} \end{array}\right)}^{T}$ associated with ω and *k* to satisfy Equations (4), (7) and (8) at the same time.

To solve these equations, we can fix ω, and search for *k* and q. At angular frequency ω, the dynamic equation of a unit cell writes

(9) $$(-\omega^{2}M+K)q=Hq=f$$

After condensing all the internal DOFs at frequency ω, it gives

(10) $$\left[\begin{array}{cc} D_{\mathrm{LL}} & D_{\mathrm{LR}}\\ D_{\mathrm{RL}} & D_{\mathrm{RR}} \end{array}\right]\left(\begin{array}{c} q_{L}\\ q_{R} \end{array}\right)=\left(\begin{array}{c} f_{L}\\ f_{R} \end{array}\right)$$

where

(11) $$\left[\begin{array}{cc} D_{\mathrm{LL}} & D_{\mathrm{LR}}\\ D_{\mathrm{RL}} & D_{\mathrm{RR}} \end{array}\right]=\left[\begin{array}{cc} H_{\mathrm{LL}} & H_{\mathrm{LR}}\\ K_{\mathrm{LR}}^{H} & H_{\mathrm{RR}} \end{array}\right]-\left[\begin{array}{cc} H_{\mathrm{LI}} & H_{\mathrm{LE}}\\ H_{\mathrm{RI}} & H_{\mathrm{RE}} \end{array}\right]{\left[\begin{array}{cc} H_{\mathrm{II}} & H_{\mathrm{IE}}\\ H_{\mathrm{EI}}^{T} & H_{\mathrm{EE}} \end{array}\right]}^{-1}{\left[\begin{array}{cc} H_{\mathrm{LI}} & H_{\mathrm{LE}}\\ H_{\mathrm{RI}} & H_{\mathrm{RE}} \end{array}\right]}^{T}$$

Introducing condition (7) and (8) into Equation (10), and eliminating $f_{L}$ and $f_{R}$, it comes to the following eigenvalue problem

(12) $$\left(\left[\begin{array}{cc} 0 & I\\ -D_{\mathrm{RL}} & -D_{\mathrm{RR}} \end{array}\right]-\lambda \left[\begin{array}{cc} I & 0\\ D_{\mathrm{LL}} & D_{\mathrm{LR}} \end{array}\right]\right)\left(\begin{array}{c} q_{L}\\ q_{R} \end{array}\right)=0$$

Eigenvalue problem (12) yields all the wavenumbers and associated deformations (termed wave shape) that can occur in the waveguide at the given frequency. Repeating the calculation in different frequencies the dispersion curves can be obtained. The required energy terms in (3) can be calculated from the wave shapes, and this will be further discussed in Section 4.1.

### 3.2. Reduced Model for the Piezoelectric Composites

Though WFEM is more efficient that the conventional finite element method in terms of wave-dispersion and forced response analysis at high frequencies, it also requires long computational time if the internal DOFs are enormous. The main reason is that in Equation (11), a matrix inverse is required at each frequency. The computational efficiency significantly decreases with the increase of matrix dimension. This corresponds to the situation where the waveguide has complicate configuration on each unit cell. In this paper, the computing of energies are required as shown in (3), this amplifies the computational cost. To accelerate the computing, we employ the reduce model proposed by the authors [31,36] because it is proved applicable for piezoelectric structures.

First, we define the transformation relation

(13) $$q=\left(\begin{array}{c} q_{L}\\ q_{R}\\ q_{I}\\ q_{E} \end{array}\right)=\left[\begin{array}{cccc} I & 0 & 0 & 0\\ 0 & I & 0 & 0\\ -K_{\mathrm{II}}^{-1}K_{\mathrm{IL}} & -K_{\mathrm{II}}^{-1}K_{\mathrm{IR}} & \Psi & -K_{\mathrm{II}}^{-1}K_{\mathrm{IE}}\\ 0 & 0 & 0 & I \end{array}\right]\left(\begin{array}{c} q_{L}\\ q_{R}\\ y\\ q_{E} \end{array}\right)=T_{c}q_{c}$$

where $\Psi =\left[\begin{array}{cccc} \psi_{1} & \psi_{2} & \cdots & \psi_{l} \end{array}\right]$, and $\psi_{k}$ is the *k*th natural mode of the unit cell with $q_{L}=q_{R}=q_{E}=0$ and the corresponding natural frequencies is $\omega_{k}$. Namely, $\psi_{k}$ and $\omega_{k}$ with k=1,2,⋯,l are obtained as the eigenvectors and eigenvalues of

(14) $$\left(K_{\mathrm{II}}-\omega^{2}M_{\mathrm{II}}\right)\psi =0$$

Only *l* modes are kept in Ψ, and the number is less than that of $q_{I}$. The criterion for the selection of the retained modes is $\omega_{k}<5\omega_{m}$ where $\omega_{m}$ is the maximum excitation frequency to be considered. After introducing Equation (13) into Equation (4), the dynamic equations of a unit cell become:

(15) $$\tilde{M}\left(\begin{array}{c} {\ddot{q}}_{L}\\ {\ddot{q}}_{R}\\ \ddot{y}\\ {\ddot{q}}_{E} \end{array}\right)+\tilde{K}\left(\begin{array}{c} q_{L}\\ q_{R}\\ y\\ q_{E} \end{array}\right)=\left(\begin{array}{c} f_{L}\\ f_{R}\\ 0\\ 0 \end{array}\right)$$

where

(16) $$\tilde{M}=T_{c}^{T}MT_{c}$$

(17) $$\tilde{K}=T_{c}^{T}KT_{c}$$

After these operations, the internal mechanical DOFs are transformed from $q_{I}$ to y, and the dimension of y is much smaller than $q_{I}$. In this way, the computing of Equation (11) is accelerated. Moreover, the technique also reduces the size the of wave shape, this is especially useful for the computing of the energies as it will be shown in Section 5.

## 4. Energy Formulas of WEMCF

### 4.1. Two Energy Formulas

Suppose a propagating wave exists in the OC status with wave shape $\phi_{\mathrm{OC}}={\left(\begin{array}{cccc} \phi_{L}^{T} & \phi_{R}^{T} & \phi_{I}^{T} & \phi_{E}^{T} \end{array}\right)}^{T}$, wavenumber *k* and frequency $\omega_{\mathrm{OC}}$. Two specific energy formulas of Equation (3) are proposed, as

(18) $$K_{1f}^{2}=\frac{W_{\mathrm{free}}}{V}$$

and

(19) $$K_{1b}^{2}=\frac{W_{\mathrm{block}}}{V}$$

where *V* is the mechanical potential energy, $W_{\mathrm{free}}$ is the electric potential energy stored in the free intrinsic capacitance (the capacitance when imposing $f_{L}=f_{R}=f_{I}=0$) and $W_{\mathrm{block}}$ is the electric potential energy stored in the block capacitance $C_{p}$. These energy terms can be obtained by the waveshapes and matrices of the unit cell:

(20) $$V={\left(\phi_{\mathrm{OC}}^{*}\right)}^{H}K\phi_{\mathrm{OC}}^{*}$$

(21) $$W_{\mathrm{free}}=\phi_{E}^{H}\left(C_{p}+P^{T}G^{-1}P\right)\phi_{E}$$

(22) $$W_{\mathrm{block}}=\phi_{E}^{H}C_{p}\phi_{E}$$

where $\phi_{\mathrm{OC}}^{*}={\left(\begin{array}{cccc} \phi_{L}^{T} & \phi_{R}^{T} & \phi_{I}^{T} & 0 \end{array}\right)}^{T}$ for the simplification of equations. Both $K_{1f}^{2}$ and $K_{1b}^{2}$ are the approximations of $K_{W}^{2}$ based on different assumptions. The demonstrations are given as follows.

### 4.2. Demonstrations

#### 4.2.1. Relationship between the Wavenumber and Wave Shape

Periodic conditions in Equations (7) and (8) can be rewritten into matrix forms, as

(23) $$q=\left(\begin{array}{c} q_{L}\\ q_{R}\\ q_{I}\\ q_{E} \end{array}\right)=\left[\begin{array}{ccc} I & 0 & 0\\ \lambda I & 0 & 0\\ 0 & I & 0\\ 0 & 0 & I \end{array}\right]\left(\begin{array}{c} q_{L}\\ q_{I}\\ q_{E} \end{array}\right)=T_{b}\hat{q}$$

and

(24) $$\left[\begin{array}{cccc} I & \lambda^{-1}I & 0 & 0\\ 0 & 0 & I & 0\\ 0 & 0 & 0 & I \end{array}\right]\left(\begin{array}{c} f_{L}\\ f_{R}\\ f_{I}\\ f_{E} \end{array}\right)=T_{e}f=\left(\begin{array}{c} 0\\ 0\\ 0 \end{array}\right)$$

Introducing these two transformations to Equation (4), by per-multiplying $T_{e}$ and multiplying $T_{b}$ at both sides of the equation, we have

(25) $$-\omega^{2}\hat{M}\hat{q}+\hat{K}\hat{q}=0$$

where

(26) $$\hat{M}=T_{e}MT_{b}$$

(27) $$\hat{K}=T_{e}KT_{b}$$

This shows that the wave shape in the form of $\hat{q}={\left(\begin{array}{ccc} q_{L}^{T} & q_{I}^{T} & q_{E}^{T} \end{array}\right)}^{T}$ is also the eigenvector of Equation (25), and its associated eigenvalue is $\omega^{2}$ where ω is the propagating frequency of the wave at wavenumber *k*. In practice Equation (25) provides another mean to calculate the dispersion curves of the waves [35], sometimes termed the ‘direct form’ [38]. Here we use these equations in the demonstration as useful properties between wave modal shape and the corresponding wavenumber.

For a propagating wave, the wavenumber *k* is a real number therefore λ is a complex value with amplitude 1. Consequently $T_{b}$ in Equation (23) is the conjugate transpose of $T_{e}$ in Equation (24), namely

(28) $$T_{b}=T_{e}^{H}$$

Introducing the OC eigenvector $\hat{q}={\hat{\phi }}_{\mathrm{OC}}={\left(\begin{array}{ccc} \phi_{L}^{T} & \phi_{I}^{T} & \phi_{E}^{T} \end{array}\right)}^{T}$ into Equation (25) and multiplying both sides of the equation by ${\hat{\phi }}_{\mathrm{OC}}^{H}$, it gives

(29) $$\omega_{OC}^{2}=\frac{{\hat{\phi }}_{\mathrm{OC}}^{H}\hat{K}{\hat{\phi }}_{\mathrm{OC}}}{{\hat{\phi }}_{\mathrm{OC}}^{H}\hat{M}{\hat{\phi }}_{\mathrm{OC}}}$$

and according to Equations (26)–(28), we know align* HOC**K**OC = HOC**K**OC

HOC**M**OC = HOC**M**OC leading to

(30) $$\omega_{OC}^{2}=\frac{{\hat{\phi }}_{\mathrm{OC}}^{H}\hat{K}{\hat{\phi }}_{\mathrm{OC}}}{{\hat{\phi }}_{\mathrm{OC}}^{H}\hat{M}{\hat{\phi }}_{\mathrm{OC}}}=\frac{\phi_{\mathrm{OC}}^{H}K\phi_{\mathrm{OC}}}{\phi_{\mathrm{OC}}^{H}M\phi_{\mathrm{OC}}}$$

This means that once the wave shape is known, we will also know the associated wavenumber. Equation (30) shows such a relation in OC status, later we will also do this in SC status. However, we will not use the ‘real’ SC wave shape to calculate SC propagating frequency but use the information in OC status to have a good guess of the SC wave shape and use it to estimate the SC propagating frequency.

#### 4.2.2. Demonstration of Energy Formula $K_{1b}^{2}$

For SC situation, if we assume that mechanical deformation under a same wavenumber *k* remains the same, namely

(31) $$\phi_{\mathrm{SC},1}={\left(\begin{array}{cccc} \phi_{L}^{T} & \phi_{R}^{T} & \phi_{I}^{T} & 0 \end{array}\right)}^{T}$$

and introduce ${\hat{\phi }}_{\mathrm{SC},1}={\left(\begin{array}{ccc} \phi_{L}^{T} & \phi_{I}^{T} & 0 \end{array}\right)}^{T}$ into Equation (25) and multiplying both sides of the equation by ${\hat{\phi }}_{\mathrm{SC},1}^{H}$, it gives

(32) $$\omega_{SC,1}^{2}=\frac{{\hat{\phi }}_{\mathrm{SC},1}^{H}\hat{K}{\hat{\phi }}_{\mathrm{SC},1}}{{\hat{\phi }}_{\mathrm{SC},1}^{H}\hat{M}{\hat{\phi }}_{\mathrm{SC},1}}=\frac{\phi_{\mathrm{SC},1}^{H}K\phi_{\mathrm{SC},1}}{\phi_{\mathrm{SC},1}^{H}M\phi_{\mathrm{SC},1}}$$

where $\omega_{SC,1}$ is an approximated frequency for the SC case. Please note that M matrix has zero terms in the lines correspond to the electric DOFs, shown in Equation (5), it makes

(33) $$\phi_{\mathrm{OC}}^{H}M\phi_{\mathrm{OC}}=\phi_{\mathrm{SC},1}^{H}M\phi_{\mathrm{SC},1}$$

According to Equations (6) and (31), there is

(34) $$\phi_{\mathrm{OC}}^{H}K\phi_{\mathrm{OC}}=\phi_{\mathrm{SC},1}^{H}K\phi_{\mathrm{SC},1}+\phi_{E}^{H}C_{p}\phi_{E}$$

Introducing Equations (30) and (32) into (2) and using (33) and (34), we obtain

(35) $$\frac{\omega_{\mathrm{OC}}^{2}-\omega_{\mathrm{SC},1}^{2}}{\omega_{\mathrm{SC},1}^{2}}=\frac{\phi_{E}^{H}C_{p}\phi_{E}}{{\left(\phi_{\mathrm{OC}}^{*}\right)}^{H}G\phi_{\mathrm{OC}}^{*}}$$

The right-hand-side of Equation (35) is exactly the same as Equation (19). The left side of Equation (35) is an approximation of Equation (2). In this regard, we show that

(36) $$K_{W}^{2}\approx K_{1b}^{2}$$

which means that $K_{1b}^{2}$ is an approximation of $K_{W}^{2}$ with the assumption (31).

#### 4.2.3. Demonstration of Energy Formula $K_{1f}^{2}$

The core of the demonstration in the previous section is to guess the SC wave shape based on the OC wave shape, and it was assumed that the mechanical deformation under a same wavenumber *k* remains the same in the SC and OC situations. More accurately, we can remove the static contribution of the OC voltage from the mechanical field so as to approximate the SC wave shape $\phi_{\mathrm{SC},2}$. Namely,

$$\begin{array}{cc} \phi_{\mathrm{SC},2} & =\phi_{\mathrm{OC}}-(\begin{array}{c} G^{-1}P\phi_{E}\\ \phi_{E} \end{array})\\ & =\phi_{\mathrm{SC},1}-(\begin{array}{c} G^{-1}P\phi_{E}\\ 0 \end{array}) \end{array}$$

Similarly, the following statement holds

(37) $$\omega_{SC,2}^{2}=\frac{{\hat{\phi }}_{\mathrm{SC},2}^{H}\hat{K}{\hat{\phi }}_{\mathrm{SC},2}}{{\hat{\phi }}_{\mathrm{SC},2}^{H}\hat{M}{\hat{\phi }}_{\mathrm{SC},2}}=\frac{\phi_{\mathrm{SC},2}^{H}K\phi_{\mathrm{SC},2}}{\phi_{\mathrm{SC},2}^{H}M\phi_{\mathrm{SC},2}}$$

and with the fact that

(38) $$\phi_{\mathrm{OC}}^{H}K\phi_{\mathrm{OC}}=\phi_{\mathrm{SC},2}^{H}K\phi_{\mathrm{SC},2}+\phi_{E}^{H}\left(C_{p}+P^{T}G^{-1}P\right)\phi_{E}$$

we can give another approximation of $K_{1}^{2}$, as

(39) $$\frac{\omega_{\mathrm{OC}}^{2}-\omega_{\mathrm{SC},2}^{2}}{\omega_{\mathrm{SC},2}^{2}}=\frac{\phi_{E}^{H}\left(C_{p}+P^{T}G^{-1}P\right)\phi_{E}}{{\left(\phi_{\mathrm{OC}}^{*}\right)}^{H}G\phi_{\mathrm{OC}}^{*}}$$

Comparing this Equation with Equations (2) and (18), it leads to

(40) $$K_{W}^{2}\approx K_{1f}^{2}$$

which means that $K_{1f}^{2}$ is also an approximation of $K_{W}^{2}$ with the assumption (38).

### 4.3. Implementations

**Figure 3:**
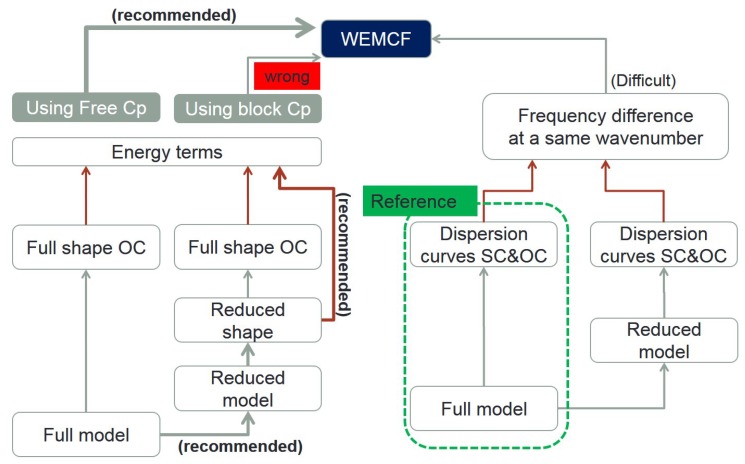
Illustration of different paths for the calculation of WEMCF in the framework of WFEM.

We proposed three different ways to calculate WEMCF: one frequency formula (2) and two energy formulas (18) and (19). Since the WFEM can be implemented by the full or reduced models, there are even more paths to implement WEMCF, as shown in Figure 3. For the calculation of $K_{W}^{2}$, there are two ways to calculate the required SC and OC frequencies. For the $K_{1f}^{2}$ and $K_{1b}^{2}$ that are based on energy terms, the wave shapes are required. To do that three paths are possible: the full shapes obtained by full WFEM which is the slowest, the full shapes obtained by reduced WFEM and the reduced shapes obtained by reduced WFEM. In combine there are 8 different ways to implement WFEM: 3 for $K_{1f}^{2}$, 3 for $K_{1b}^{2}$ and another 2 for $K_{W}^{2}$. In the next section we will examine the efficiency and accuracy of the implementations, and finally give the best path.

## 5. Validations

### 5.1. Validation of the Implementations

**Figure 4:**
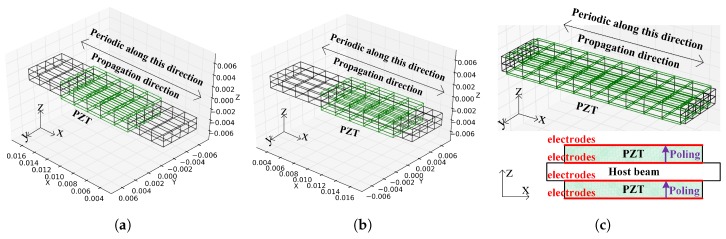
Unit cells of the piezoelectric waveguides: (**a**) unit cell A; (**b**) unit cell B which is a non-symmetric way of choosing the unit cell for the infinite periodic structure with unit cell A; (**c**) unit cell C which has longer PZT patches. The Piezoelectric materials are polarized along the z axis, and the electrodes fully cover the surfaces parallel to the x-y plane (orthogonal to the polarized direction). The program code used to generate the Finite Element models in ANSYS can be found in the Supplementary Files.

**Figure 5:**
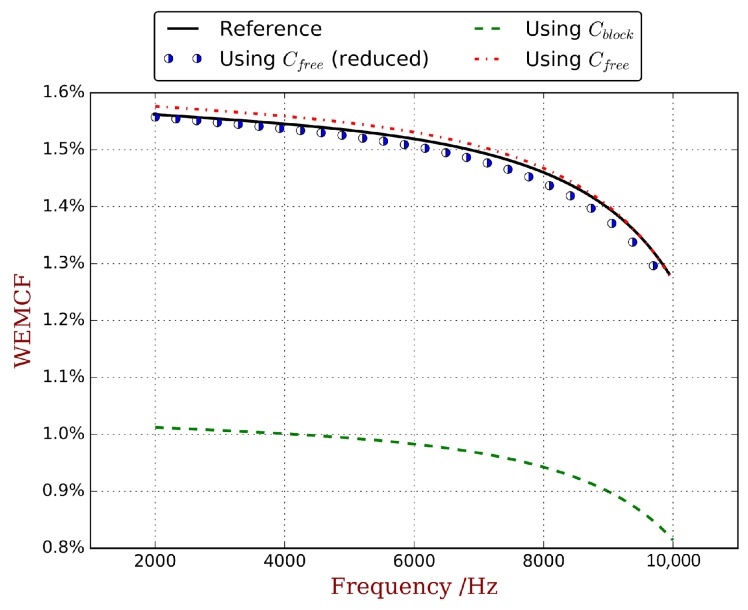
Comparison of WEMCF calculated by using: full WFEM with $K_{W}$ (labelled by ‘Reference’), full WFEM with $K_{1b}$ (labelled by ‘using $C_{\mathrm{block}}$’), full WFEM with $K_{1f}$ (labelled by ‘using $C_{\mathrm{free}}$’) and reduced waveshape with $K_{1f}$ (labelled by ‘using $C_{\mathrm{free}}$ (reduced)’).

As mentioned, the consistency of WEMCF with MEMCF is strongly desired, and $K_{W}^{2}$ is consistent with MEMCF. Therefore the $K_{W}^{2}$ calculated by full WFEM is regarded as the reference. The piezoelectric waveguide with unit cell A shown in Figure 4a is considered. The mesh quality of these models are verified by the forced response analysis [31]. The host material is steel without damping, with a Young’s modulus of E=2.11d11Pa, a Poisson’s ratio of 0.3 and a density of $7.8d3kg/m^{3}$. The used piezoelectric material is PZT4 whose material parameters are listed in the Appendix A. The WEMCF for the z-transverse wave from 2e3 Hz to 1e4 Hz is computed through all 8 paths listed in Figure 3, and the highlighted results are compared in Figure 5. The main observation is that all 3 paths for $K_{1b}^{2}$ have significant errors, even though the overall tendencies are the same as the reference. The other 5 paths all return acceptable results with relative error less than 3%. This may due to the fact that assumption (38) is more precise than (31).

**Figure 6:**
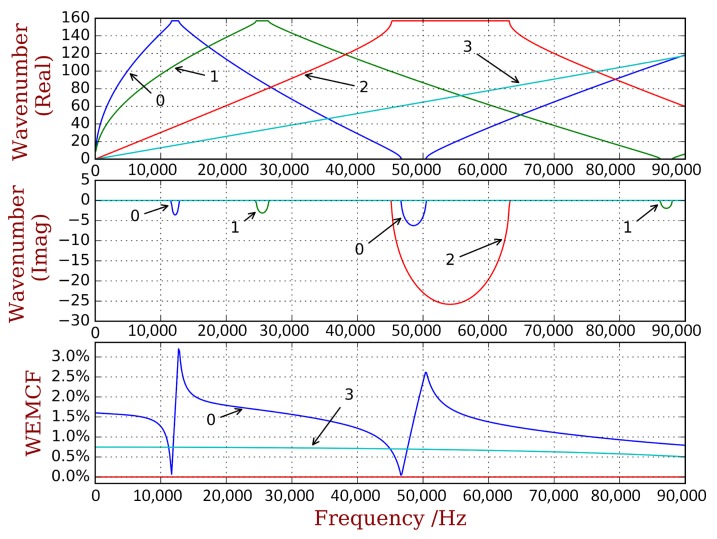
Dispersion curves and WEMCF for the piezoelectric waveguide with unit cell A: only wave 0 (z transverse) and 3 (longitudinal) have significant values.

**Figure 7:**
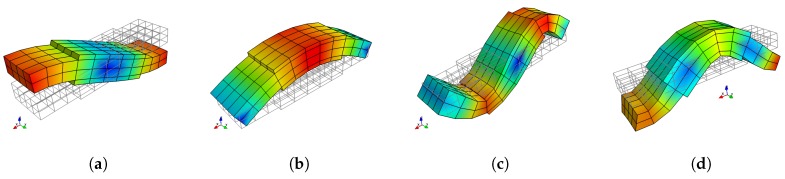
Waveshape of wave 0 of unit cell A at the border frequencies of the band gaps: (**a**) 11.634 kHz, low WEMCF; (**b**) 12.556 kHz, high WEMCF; (**c**) 46.475 k Hz, low WEMCF; (**d**) 50.077 kHz, high WEMCF.

A complete dispersion curves and WEMCF results for the piezoelectric periodic structure with unit cell A is shown in Figure 6. It is shown that the z-axis flexural wave (marked by number 0) has the most significant electromechanical coupling. Weaker WEMCF is observed for the longitudinal wave (3). No coupling effects are reported for the torsional (2) and y-axis flexural waves (1). These conclusions can be acknowledged by the geometric configuration of the unit cell and engineering common sense. It is interesting to see that the largest and lowest values emerge at the border frequencies of the band gaps. The wave shapes of these waves are presented in Figure 7. The symmetric wave shapes generates different kind of charges on the electrode, cancelling each other, leading to a very low WEMCF. On the contrary, anti-symmetric shapes always generate same kind of charges hence maximize the WEMCF.

**Figure 8:**
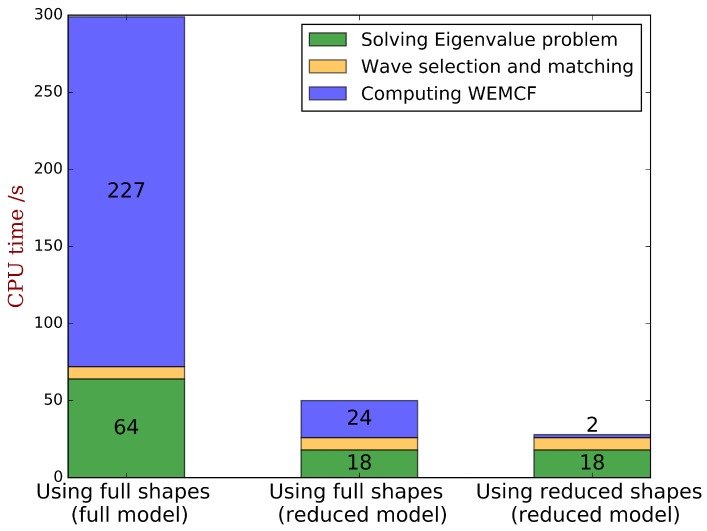
CPU time for the full dispersion characteristics with WFEM of unit cell A by the energy formula $K_{1f}^{2}$ where the energies are calculated by: (1) using full shapes obtained from full WFEM; (2) using full shapes obtained from reduced WFEM; (3) using reduced shapes. The label ’Wave selection and matching’ refers to the standard post-processing of the WFEM results in order to identify the same type of wave among different frequencies [36], not the matching between the results between the OC and SC status as required for computing the frequency formula of WEMCF.

As discussed, among all the valid paths, the ones use $K_{W}^{2}$ are difficult to be programmed for general cases. There remains 3 valid paths for $K_{1f}^{2}$: (1) using full shapes obtained from the full WFEM; (2) using full shapes obtained from the reduced WFEM; (3) using the reduced wave shapes. The CPU time for obtaining the full dispersion characteristics with WFEM of unit cell A are compared in Figure 8. It shows that the post-processing for WEMCF for the full WFEM is a heavy task, and the CPU time is nearly 3 times of the free-wave analysis. While the reduced unit cell model is used, the computing time for WEMCF is reduced even when using the full wave shapes. The CPU time has the same magnitude as the free-wave analysis in this case, because the reduced model can accelerate the mapping from the cross-section DOFs to the internal DOFs by avoiding the matrix inverse. Only minor additional time is required when reduced shapes are used to compute the energy terms for WEMCF, it reduces the post-processing by 99% in comparison to the full WFEM.

The infinite periodic structure with unit cell A can also be represented by an unsymmetrical unit cell B, shown in Figure 4b. As discussed in the literature [39], the dispersion curves should be the same as unit cell A. We also found that the WEMCF results remain the same as well. The minimal and maximal WEMCF still happen at the border frequencies of band gaps. While using the unsymmetrical unit cell, the wave shape of a unit cell is no longer symmetric, but the deformation on the PZT patches are still symmetric or anti-symmetric, leading to zero or maximum WEMCF. To make the paper more concise, the results associated with unit cell B are not presented.

**Figure 9:**
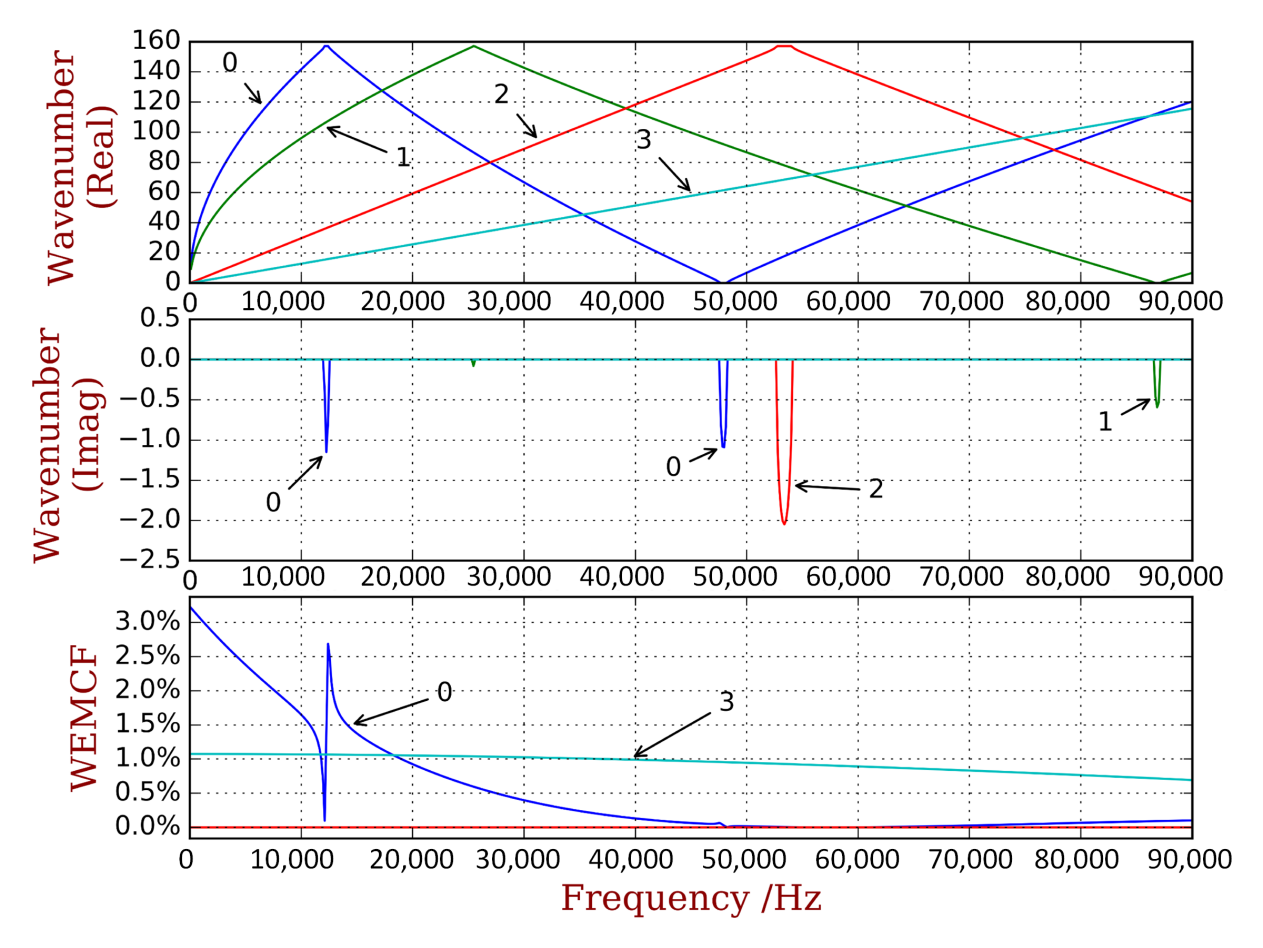
Dispersion curves and WEMCF for the piezoelectric waveguide with unit cell C.

**Figure 10:**
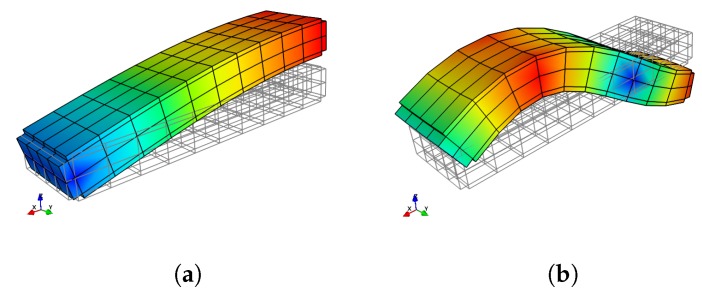
Waveshape of wave 0 of unit cell C: (**a**) at the first propagating zone; (**b**) at the second propagating zone.

Finally, we analyze the WEMCF of PZT waveguide with unit cell C, shown in Figure 4c. The unit cell has the same length as unit cell A and B but with longer PZT patches. The results are shown in Figure 9, where we observe a better WEMCF in lower frequencies than higher ones. This can be explained by checking the wave shapes shown in Figure 10. It can be seen that in higher frequencies the charges generated by the deformation will start to cancel each other. The comparison between unit cell A and C also acknowledges the engineering common sense that long PZT path works poorly in high frequencies.

### 5.2. Validation against a Thin-Wall Piezoelectric Structure

In the previous sections, we show that $K_{1f}^{2}$ is a proper way to define the WEMCF because it is a good approximation of the $K_{W}^{2}$ which is consistent with MEMCF. Using the reduced wave shape associated with the reduced model in WFEM to compute the required energies is a very efficient implementation towards $K_{1f}^{2}$, as marked in Figure 3. In this section, we use a more complex piezoelectric structure to further examine such findings.

**Figure 11:**
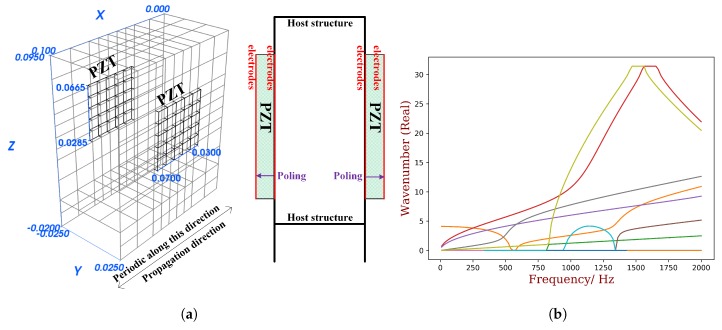
The unit cell of the thin-wall piezoelectric structure (**a**) and its dispersion curves (**b**). The program code used to generate the Finite Element model in ANSYS can be found in the Supplementary Files.

The considered thin-wall structure is shown in Figure 11a, with mesh quality verified by the forced response analysis [36]. Piezoelectric patches are periodically bonded and for each unit cell there are two PZT patches. The unit cell is then modeled by FEM, and for one single unit cell, the overall number of DOFs is 1896, with 336 on the left and right side, 1558 on the internal mechanical part and 2 on the electric part. The geometric parameters are also labelled on the figure by international units. The host and piezoelectric materials are the same as used in the previous section.

**Figure 12:**
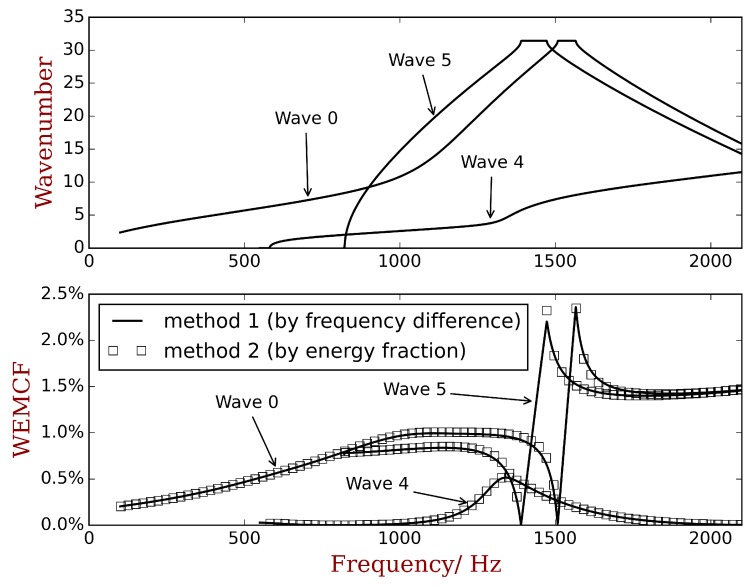
The comparison of WEMCF calculated by the frequency formula $K_{W}^{2}$ and the energy formula $K_{1f}^{2}$. Waves 0, 4 and 5 are selected from the full dispersion curves in Figure 11b. The $K_{1f}^{2}$ is calculated by the reduced wave shapes as recommended in the last section.

**Figure 13:**
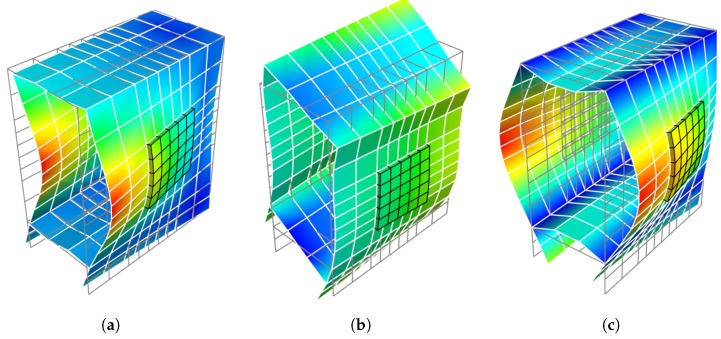
Wave shapes of the at 1000 Hz: (**a**) wave 0; (**b**) wave 4; (**c**) wave 5.

The dispersion curves in OC status are calculated from 0 to 2000 Hz by the full unit cell model, and the positive-going propagating waves are recognized and shown in Figure 11b. To obtain $K_{W}^{2}$ by definition, another calculation with SC status should be conducted, and the waves should be matched between OC and SC results. This is very difficult to programme as a general numerical tool. Here we select 3 waves for comparison, including both low-order (wave 0) and high-order waves (waves 4 and 5), and their $K_{W}^{2}$ results are computed by manually matching the waves. The $K_{1f}^{2}$ results are computed by the reduced shapes associated with the reduced model as recommended in the previous section by the in-house Python code. The comparison of the results are shown in Figure 12 where a very good agreement is observed. The results acknowledge the statement that $K_{1f}^{2}$ is a good approximation of $K_{W}^{2}$ and therefore consistent with MEMCF. The selected wave shapes are illustrated in Figure 13. The results verifies the use of WEMCF ($K_{1f}^{2}$) for complex waveguides where complex wave shapes exist.

## 6. Application: Designing the Resistive PZT Waveguide

**Figure 14:**
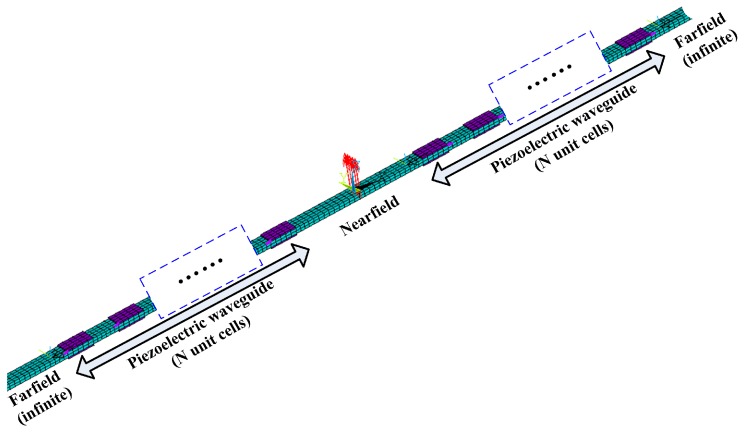
The considered built-up structure and the dividing of substructures.

To illustrate the usage of the WEMCF, let us consider a built-up structure constructed by bonding 2N groups of co-located piezoelectric patches onto a uniform host structure. *N* groups of piezoelectric patches are periodically distributed at the right side of the excitation while *N* other groups are located on the other side. The structure is infinite both to the left and right side. The FEM/WFEM hybrid method [31] can be used to analyse the energy flow and forced response. To do that, this structure is divided into five parts: one nearfield part, two piezoelectric waveguides and two uniform far-field waveguides, as shown in Figure 14.

Here we fix N=21. the external forces are applied in $u_{z}$ DOF of all the nodes that are located in the origin cross-section of the nearfield (x=0) with an amplitude of 1N. The energy flow can be attenuated by shunting resistors to the PZT patches and this effects can be quantified by the power Transmission Loss (TL):

(41) $$\mathrm{TL}=10log_{10}\left(\frac{P_{\mathrm{in}}}{P_{\mathrm{out}}}\right)$$

**Figure 15:**
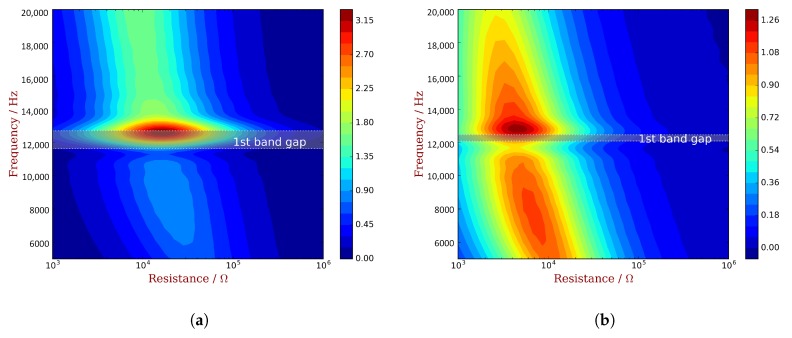
Parametric study of power transmission loss with respect to frequency and resistance, when the piezoelectric waveguides use: (**a**) unit cell A; (**b**) unit cell C.

We conduct the parametric studies of TL with respect to the frequency and resistance. PZT waveguides with unit cell A and C are considered respectively, and the results are shown in Figure 15. It can be seen that the best TL and the associated resistance vary with frequency. Results also indicate some correlation between the best TL and the WEMCF: unit cell A has a better WEMCF in the second propagating zone; and the best TL is larger in the second propagating zone than in the first one, shown in Figure 15a; unit cell C has better WEMCF in the first propagating zone than the second, and a lower best TL can be observed in higher frequencies.

**Figure 16:**
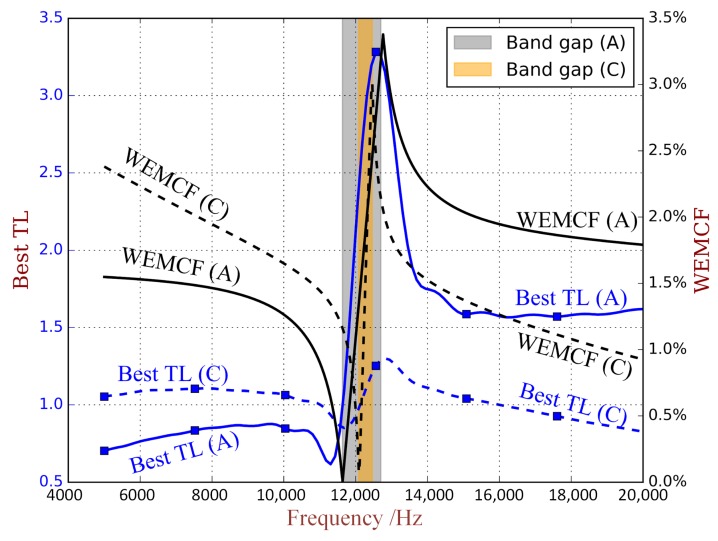
The best TL and WEMCF with respect to the frequency for PZT waveguide with unit cell A and unit cell C.

To illustrate this correlation more clearly, we plot the best TL with respect to the frequency when using unit cells A and C in Figure 16, in association with the WEMCF of wave 0 of unit cell A and C. In the first propagating zone, we can see that unit cell A has stronger WEMCF than C, and the best TL when using A is better than C. In the second propagating zone, we can see that unit cell A has weaker WEMCF than C, and the best TL when using A is weaker than C.

This correlation between WEMCF and best TL allows to compare configurations of unit cell without performing the forced response and energy analysis of the built-up structure. It means the design for the geometric and electric parameters can be done separately: first we determine the geometric parameters so as to achieve the best WEMCF, only by free-wave analysis; then we only consider the unit cell with optimized geometrics to determine the electric parameters so as to achieve the best power attenuation.

## 7. Conclusions

In this paper, we study the factor that allows to quantify the coupling strength between the mechanical and electric fields during the passage of a wave in piezoelectric composites, termed the wave electromechanical coupling factor (WEMCF). We show that to maintain the consistency with the classical modal electromechanical coupling factor (MEMCF), the WEMCF should also be defined by the frequency difference of the OC and SC statues. However, this definition has difficulties in calculation for general piezoelectric waveguide for the need of computing dispersion curves twice and matching waves between SC and OC statuses.

To address this challenge, an effective energy formula for WEMCF is proposed, defining the WEMCF as the percentage of electric energy stored in the free intrinsic capacitance over the mechanical energy during the passage of the wave. This energy formula is a good approximation to the frequency formula, and we only need to analyze the dispersion curves once and do not need to match waves from different electrode statuses.

The proposed energy formula can be calculated as a post-processing of the wave and finite element method. We recommend the use of reduced wave shapes for a fast calculation of WEMCF. It reduces the CPU time for computing WEMCF of the example structure to less than 1%, and it works for complex structures. Please note that the method can be used in any frequency ranges, provided that an appropriate FE model for the unit cell is established.

An application is given, concerning the energy flow attenuation in a built-up structure by resistive PZT shunts. We show that the WEMCF is strongly correlated with the best energy transmission loss. This provides more insights to understand the waveguide performance and it also allows the design of the geometric and electric parameters to be done separately. 

## Supplementary Materials

The following are available online at http://www.mdpi.com/1996-1944/11/8/1406/s1: File ‘APDL_UC_A.txt’—Code in ANSYS parametric design language (APDL) for unit cell A used in the paper. File APDL_UC_B.txt—Code in APDL for unit cell B. File ‘APDL_UC_C.txt’—Code in APDL for unit cell C. File ‘APDL_UC_Thin_wall.txt’—Code in APDL for the piezoelectric thin-wall structure.

## Abbreviations

The following abbreviations are used in this manuscript:

EMCF	electromechanical coupling factor
WEMCF	wave electromechanical coupling factor
MEMCF	modal electromechanical coupling factor
WFEM	wave and finite element method
FRF	frequency response function
TL	transmission loss
OC	open circuit
SC	short circuit
DOF	Degree-of-freedom

## Appendix A. Material Properties of the Piezoelectric Material (PZT4)

Mass density: $\rho =7500\;\mathrm{kg}/m^{3}$

Material stiffness matrix evaluated at constant electric field:

$$\left[c^{E}\right]=10^{10}\times \left[\begin{array}{cccccc} 13.2 & 7.1 & 7.3 & & & \\ 7.1 & 13.9 & 7.3 & & & \\ 7.3 & 7.3 & 11.5 & & & \\ & & & 2.6 & & \\ & & & & 2.6 & \\ & & & & & 3.1 \end{array}\right]\mathrm{Pa}$$

Permittivity matrix evaluated at constant strain:

$$\left[\epsilon^{S}\right]=\left[\begin{array}{ccc} 804.6\epsilon_{0} & & \\ & 804.6\epsilon_{0} & \\ & & 659.7\epsilon_{0} \end{array}\right]$$

where $\epsilon_{0}=8.85\times 10^{-12}\;C/\left(V\;\cdot \;m\right)$.

Piezoelectric stress coupling matrix:

$$\left[e\right]=\left[\begin{array}{ccc} & & 4.1\\ & & 4.1\\ & & -14.1\\ & -10.5 & \\ -10.5 & & \end{array}\right]N/\left(V\;\cdot \;m\right)$$
